# 

**Published:** 1902-09-13

**Authors:** 


### NOTICE TO CORRESPONDENTS.

**All MSS., Letters, Books for Review, and other matters intended for the Editor should be addressed The Editor, "The Hospital," The Hospital Building, 28 & 29 Southampton Street, Strand, W.C.**
**The Editor cannot undertake to return rejected MS., even when accompanied by stamped directed envelope.**

**Notice to Advertisers and Subscribers.**

**All Advertisements, Orders for Copies of The Hospital, and Business Communications should be addressed to THE MANAGER, (Not to the Editor), The Hospital, 28 & 29 Southampton Street, Strand, London, W.C.**
**The Cost of Subscription, post free, is as follows.—Per week, 2½d.; per quarter, 2s. 8d.; per half-year, 5s. 3d.; per annum, 10s. 6d. Foreign:—Per annum, 15s. 2d., payable, in all cases, in advance.**

**NOTICE.—Vol. XXXI. of "The Hospital" (October 1901, to March 1902), in handsome cloth binding is now on sale at the office of this Journal, price, 7s. 6d. Cases for binding the Numbers contained in this Volume 1s. 6d. Index to Vol. XXXI., 2½d., post free.**
**The Monthly Part for September is now ready. Price 6d., by post 8d.**

### THE STUDENT OF MEDICINE.

### APPOINTMENTS.

R. Alliott, M.R.C.S.Eng., L.R.C.P.Lond., appointed District Medical Officer of the Braintree Union. **B. G. Fiddian**, M.R.C.S.Eng., L.R.C.P.Lond., appointed House Surgeon to the Newport Hospital. **P. T. Finn**, L.R.C.P., L.R.C.S.Edin., appointed Certifying Factory Surgeon for the Ashwick District of Somersetshire. **Bernard Gilpin Forman**, M.B.Edin., appointed Medical Officer of Health to the Scalby Urban District Council, and District Medical Officer for the Scalby District of the Scarborough Union, and Public Vaccinator. **W. J. Hill**, L.R.C.P.Lond., M.R.C.S.Eng., appointed Medical Officer of Health for the Clevedon Urban District Council. **D. Knocker**, M.B.Lond., M.R.C.S.Eng., appointed Medical Officer of Health for Lexden. **Aylmer A. Macfarlane**, M.D., appointed Resident Medical Officer to the Parkside Lunatic Asylum, Adelaide, South Australia. **J. I. Macmillan**, M.B., M.S.Glas., appointed Certifying Factory Surgeon for the Laurencekirk District, Kincardineshire. **Mary J. Menzies**, M.B., B.S.Edin., appointed Assistant Resident Medical Officer to the Birkenhead Workhouse Infirmary. **W. Rees**, M.R.C.S., L.R.C.P.Lond., appointed Medical Officer of Health to the Llandilo Urban District Council. **E. A. Sadler**, M.D.Lond., M.R.C.S.Eng., appointed Medical Officer of Health to the Ashbourne Rural District Council. **J. H. Stewart**, L.R.C.P., L.R.C.S.Edin., appointed Certifying Factory Surgeon for the Cruden District of Aberdeenshire. **I. H. L. Tyleiste**, M.B., Ch.B.Vict., appointed Chief Medical Officer to the Gold Coast Government Railways. **H. M. Williamson**, M.B., Ch.B.Vict., appointed Assistant Medical Officer to the Chorlton Union Workhouse.

ST. THOMAS'S HOSPITAL.—The following gentlemen have been selected as House Officers from Tuesday, September 2nd, 1902:—House Physicians—**A. D. Hamilton**, M.B.Lond., L.R.C.P., M.R.C.S.; **C. H. Sedgwick**, M.A., M.B., B.C., Cantab. Assistant House Physicians—**W. H. Harwood Yarred**, B.Sc.Lond., L.R.C.P., M.R.C.S.; **A. Mavrogordato**, L.R.C.P., M.R.C.S. House Surgeons—**W. Hill**, B.A.Cantab., L.R.C.P., M.R.C.S.; **J. Coates**, L.R.C.P., M.R.C.S.; **A. C. Hudson**, M.A., M.B., B.C.Cantab.; **H. Spurrier**, B.A.Cantab., L.R.C.P., M.R.C.S. Assistant House Surgeons—**J. W. Rob**, B.A., M.B., B.C.Cantab.; **T. B. Henderson**, M.A., M.B., B.Ch.Oxon.; **J. P. Hedley**, M.A., B.C.Cantab.; **A. B. Bradford**, L.R.C.P., M.R.C.S. Obstetric House Physicians—(*Senior*) **R. E. Roberts**, M.B., B.Sc.Lond.; (*Junior*) **F. J. Child**, M.A., M.B., B.C.Cantab., L.R.C.P., M.R.C.S. Ophthalmic House Surgeons—(*Senior*) **F. Clarkson**, M.B., B.S.Durh.; (*Junior*) **A. E. A. Loosely**, B.A.Oxon., L.R.C.P., M.R.C.S. Clinical Assistants in the Special Department for Diseases of the Throat—**J. E. Adams**, L.R.C.P., M.R.C.S.; **S. G. Scott**, M.A., M.B., B.Ch.Oxon. Clinical Assistants in the Special Department for Diseases of the Skin—**A. T. Waterhouse**, M.A., M.B., B.Ch.Oxon., L.R.C.P., M.R.C.S.; **C. Wheen**, B.A.Oxon., L.R.C.P., M.R.C.S. Clinical Assistant in the Special Department for Diseases of the Ear—**F. H. Pearce**, B.A., L.R.C.P., M.R.C.S. Clinical Assistant in the Electrical Department—**O. Hildesheim**, B.A., M.B., B.Ch.Oxon.

### "The Hospital" Institutional Library.

"Hospitals and Asylums of the World." 4 Vols., with a Portfolio of Plans, complete £12 12s.

"Burdett's Hospitals and Charities, 1902." 5s.

"Cottage Hospitals, General, Fever, and Convalescent." 10s. 6d.

"Hospital Expenditure: The Commissariat." 2s. 6d.

"A Legal Handbook for the Use of Hospital Authorities." 2s. 6d.

"The Cottage Hospital Case Book or Register of Patients." 10s. and 12s. 6d.

"The Furnishing and Appliances of a Cottage Hospital." 6d.

All these are published by the SCIENTIFIC PRESS, Ltd., and may be obtained through any bookseller, or direct from the publishers, 28 and 29 Southampton Street, Strand, London, W.C.

